# From Powders to Performance—A Comprehensive Study of Two Advanced Cutting Tool Materials Sintered with Pressure Assisted Methods

**DOI:** 10.3390/ma18020461

**Published:** 2025-01-20

**Authors:** Kinga Momot, Piotr Klimczyk, Beata Leszczyńska-Madej, Marcin Podsiadło, Yuliia Rumiantseva, Agnieszka Gubernat

**Affiliations:** 1 Łukasiewicz Research Network, Krakow Institute of Technology, 30-418 Krakow, Poland; kbednarc@agh.edu.pl (K.M.);; 2 Faculty of Non-Ferrous Metals, AGH University of Krakow, 30-059 Krakow, Poland; bleszcz@agh.edu.pl; 3 Faculty of Materials Science and Ceramics, AGH University of Krakow, 30-059 Krakow, Poland; gubernat@agh.edu.pl

**Keywords:** high pressure–high temperature (HPHT), spark plasma sintering (SPS), cubic boron nitride (cBN), ceramic composites, cutting tools, Inconel 718

## Abstract

This paper presents a comprehensive study of two tool materials designed for the machining of Inconel 718 superalloy, produced through two distinct sintering techniques: High Pressure–High Temperature (HPHT) sintering and Spark Plasma Sintering (SPS). The first composite (marked as BNT), composed of 65 vol% cubic boron nitride (cBN), was sintered from the cBN–TiN–Ti_3_SiC_2_ system using the HPHT technique at a pressure of 7.7 GPa. The second composite (marked as AZW) was fabricated from the Al_2_O_3_–ZrO_2_–WC system using SPS at a pressure of 63 MPa. The final phase composition of BNT material differed significantly from the initial composition due to reactions occurred during sintering. In contrast, the phase composition of the AZW ceramic composite before and after sintering was similar. The materials exhibited high quality, as evidenced by a Young’s modulus of 580 GPa for BNT and 470 GPa for AZW, along with hardness of 26 GPa for BNT and 21 GPa for AZW. Both composites were used to prepare cutting inserts that were evaluated for their performance in machining Inconel 718 alloy. While both inserts showed durability comparable to their respective reference commercial inserts, they differed in performance and price relative to one another.

## 1. Introduction

Nickel-based superalloys, particularly Inconel 718, have become increasingly critical in the aerospace and automotive sectors due to their exceptional mechanical properties at elevated temperatures. However, machining of these alloys presents significant challenges, primarily due to their low thermal conductivity and propensity for hardening during cutting operations. Tool materials intended for cutting inserts used in machining Inconel 718 should have high hardness, mechanical strength, and wear resistance preserved at high temperatures. This is important since cutting tools are subjected to significant blade heating during cutting processes [1,2].

Ceramic matrix composites (CMCs) are of great interest because they meet almost all the requirements described above. However, their main drawback is low fracture toughness, which can often lead to catastrophic failure during use [3]. CMCs that combine oxide and carbide or nitride phases are especially promising for demanding applications, including cutting inserts used in the machining of hard-to-cut materials [4,5,6,7,8,9,10,11,12,13]. Oxide-carbide group of CMCs includes composites based on aluminium oxide with the addition SiC whiskers [8,9], titanium carbide [7,10], titanium nitride [10], as well as widia (WC-Co) [4,11] and tungsten carbide [7,10,12,13]. The combination of Al_2_O_3_ and ZrO_2_ with WC creates a strong synergistic effect resulting from high hardness, low abrasion, high Young’s modulus and high strength of the carbide phase, as well as high chemical corrosion resistance at elevated temperatures specific to the oxides [10,12,13,14,15]. It was found that the alumina matrix tungsten carbide composite, which contains a small addition of ZrO_2_, exhibited exceptionally high mechanical strength [13].

Another notable group of tool materials are superhard composites based on cubic boron nitride. Similar to carbon, boron nitride can exist in two forms: a graphite-like hexagonal form (hBN) and a cubic polymorph (cBN) with diamond-like properties. Since cBN is a metastable phase, its sintering requires high temperatures, typically above 2000 °C, and pressures of 5–8 GPa [16]. Composites with cBN can be generally classified as those with high (>70 vol.%) or low cubic boron nitride content. The latter, which were originally developed because of the difficulties associated with sintering pure cBN, usually contain a ceramic binder such as AlN, TiN or TiC [17,18]. This approach lowers the sintering temperature and allows for better control over the microstructure and key properties of the tool material, including mechanical, tribological, thermal, and electrical properties (the latter enabling electrical discharge machining). Cutting tools made from cBN composites with a relatively high content of ceramic bonding phase can be effectively used in advanced machining processes, including the turning of hardened steels, cast irons, and especially nickel-based superalloys [19,20]. Costes et al. highlighted the impact of cBN content, grain size, and the type of bonding phase on the lifetime of cBN cutting inserts. The results clearly demonstrate that cutting blades containing approximately 60–65% cBN exhibited longer working times and better wear resistance compared to those with higher cBN content [21].

Titanium compounds, such as TiC or TiN, which have been widely used as ceramic binders in superhard composites for years, exhibit excellent high-temperature properties and good oxidation resistance, although their ductility remains low [22]. Therefore, it is desirable to look for innovative bonding phases that combine both optimal chemical and mechanical properties. In the case of superhard diamond composites, many scientific papers address the use of so-called MAX phases [23,24,25]. MAX phases are ternary carbide-nitrides with layered crystalline structure, exhibiting properties intermediate between ceramics and metals [26]. One example of a MAX phase is Ti_3_SiC_2_, an unstable compound that decomposes at temperatures above 800 °C. As the pressure increases, the decomposition temperature of this material decreases [27]. This means that during pressure assisted sintering, the MAX phase is expected to decompose and form new compounds, which may provide a new route to in situ formation of a multi-component bonding phase in composites.

Considering the above, this study aimed to manufacture two grades of cutting tool materials intended for machining the Inconel 718 superalloy. The first grade was a superhard composite that combined 65 vol.% cBN with a new multi-component bonding phase, which was obtained in situ through the decomposition of Ti_3_SiC_2_ during sintering. The concept for the CMC involved producing a material consisting of interpenetrating oxide (Al_2_O_3_ + ZrO_2_) and carbide (WC) phases in approximately equal volume proportions, resulting in a duplex structure.

The two distinct sintering techniques employed for the fabrication of cBN–TiN–Ti_3_SiC_2_ superhard composites and Al_2_O_3_–ZrO_2_–WC ceramic composites were High Pressure–High Temperature (HPHT) sintering and Spark Plasma Sintering (SPS), respectively. This choice was dictated by the specific properties of each material.

The HPHT method is relatively expensive and not very efficient on an industrial scale, but it provides extremely high pressures, typically up to 10 GPa, and high temperatures reaching 2400 °C. As mentioned above, HPHT conditions are necessary for sintering metastable materials such as diamond or cBN, as they preserve the cubic structure and maintain exceptional hardness.

On the other hand, the SPS technique utilizes pulsed electric current and moderate uniaxial pressure, typically up to 100 MPa, to facilitate rapid densification of powders at lower temperatures compared to conventional sintering methods. This makes SPS particularly advantageous for sintering ceramic matrix composites, as it enables the achievement of high density and fine microstructure within a relatively short processing time.

## 2. Materials and Methods

### 2.1. Superhard Composite—BNT

**Figure 1:**
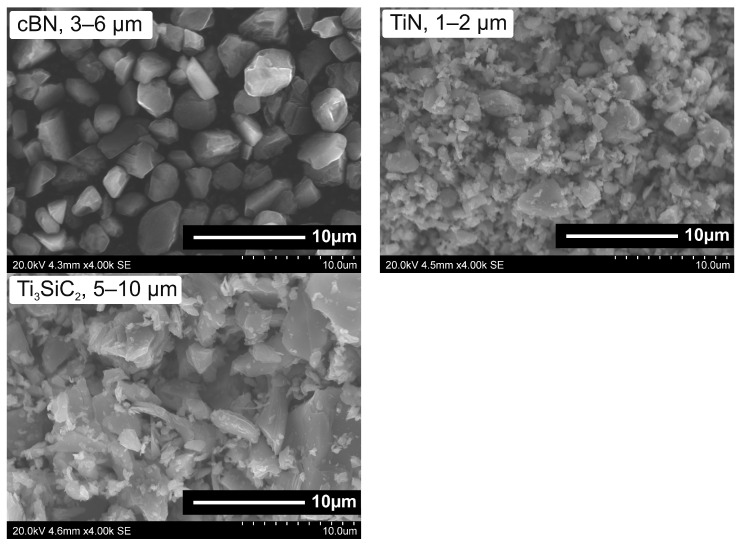
Morphology of initial powders for BNT superhard composite, SEM, ×4 k.

Cubic boron nitride (3–6 µm, Micron+ ABN M36, Element Six, Shannon, Ireland), titanium nitride (1–2 µm, C-grade, HC Starck, Goslar, Germany) and titanium silicon carbide (5–10 µm, Maxthal 312, KANTHAL, Hallstahammar, Sweden) powders were used as starting materials for preparing mixtures in the following proportion: 65% vol. cBN/20% vol. TiN/15% vol. Ti_3_SiC_2_. This composition was abbreviated as BNT after the names of the main components. Due to the fact that Ti_3_SiC_2_ powders rarely occur in pure, single-phase form, an XRD analysis was performed which showed that Maxthal 312 contains about 10 mass % of TiC. Figure 1 shows the morphology (SEM) of the powders selected for the composite mixture.

**Figure 2:**
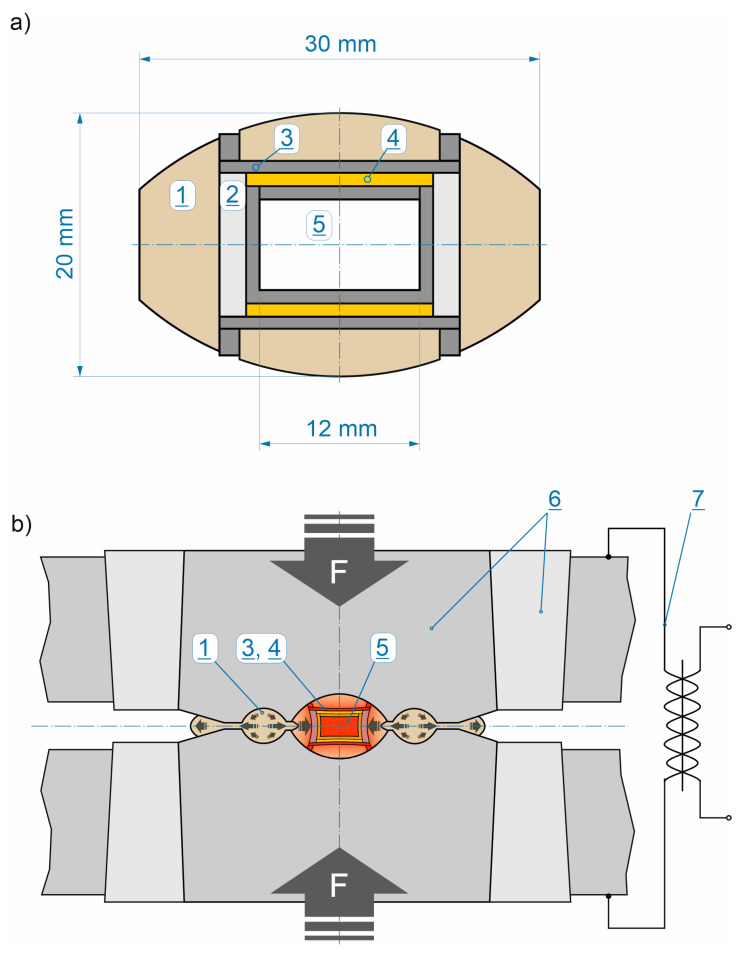
Sintering of materials in the HPHT toroidal apparatus: (**a**) Sample assembly: 1—mineral gasket, 2—hBN insulator, 3—graphite heating elements, 4—graphite + ZrO_2_ resistors (additional heating elements), 5—sample (compacted powder); (**b**) Operational diagram of the toroidal apparatus: quasi-isostatic compression of the preliminary consolidated powders (5) is achieved as a result of plastic deformation of the mineral gasket (1) between anvils (6); electrical heating of the sample is provided by a high-power current transformer (7) and heating elements (3, 4).

SEM analysis was conducted to confirm the particle sizes and shapes of commercial powders declared by suppliers. Such a basic quality control of the starting materials is important to ensure the reproducibility of the results in subsequent stages of the work. The cBN powder particles are in the size range 3–6 µm and have a polyhedral shape with sharp edges. TiN powder particles are fine (1–2 µm) and have an irregular shape. The largest particle size range was exhibited by Ti_3_SiC_2_ powder (5–30 µm). To ensure the homogeneity of the composite, all its components should be of similar size, therefore it was necessary to carry out the milling process. Powders milling and mixtures homogenisation were conducted in one single technological operation, using planetary mill (Pulverisette 6 classic line, Fritsch GmbH, Idar-Oberstein, Germany), Si_3_N_4_ milling bowl and balls and isopropyl alcohol as a wetting medium. The mixing process was conducted at a speed of 200 rpm in 3 cycles of 20 min each. Then the powder mixture was dried, sieved and compacted into special mineral-graphite assembly and sintered using a Bridgman-type HPHT toroidal apparatus (Figure 2). In this system quasi-isostatic compression of the preliminary consolidated powders is achieved as a result of plastic deformation of the mineral gasket material (usually metamorphic stones containing CaCO_3_ or/and pyrophyllite) between anvils [28,29]. Electrical heating of the sample material is provided by a high-power current transformer and graphite heater.

Composites were sintered for 60 s under 7.7 GPa and temperature range from 1600 to 2100 °C. The HPHT sintered compacts were disk-shaped with dimensions of approx. 10 mm in diameter and 4 mm in height.

### 2.2. Carbide-Oxide Ceramic Composite—AZW

Alumina powder (Al_2_O_3_-α, 100 nm, TM-DAR grade, TAIMEI, Tokyo, Japan), zirconia powder partially stabilised with yttrium oxide (ZrO_2_ + 3% mol. Y_2_O_3_, 30–60 nm, YSZ grade, Inframat, Manchester, CT, USA), and WC powder (600–700 nm, DS 60 grade, HC Starck, Goslar, Germany) were used as the starting materials. This composition was abbreviated as AZW after the chemical names of the main components.

**Figure 3:**
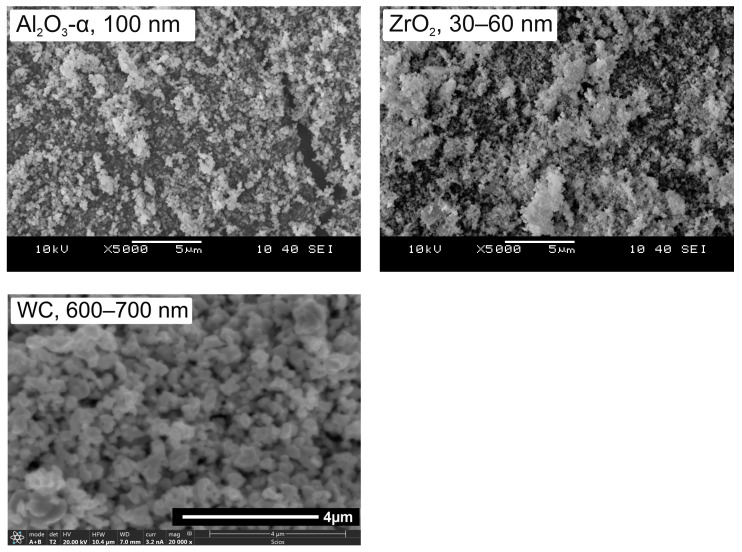
Morphology of initial powders for AZW ceramic composite, SEM, ×5 k.

Analysis of the morphology of the starting powders for AZW composites shown in Figure 3 indicates that the oxide powders are characterised by very fine particles, in the nanometric range, around 30–100 nm. The WC powder is also very fine, in the submicrometric range, with an average grain size of about 0.6 µm. These observations are in accordance with the manufacturers’ declared technical data.

Powders in volume ratios of 46:12:42 (Al_2_O_3_:ZrO_2_:WC) were mixed using Planetary Ball Mill (Pulverisette 6 classic line, Fritsch GmbH) and WC-Co milling aids. The mixing process was conducted at a speed of 200 rpm within 2 h in the isopropyl alcohol as a wetting medium. The prepared mixture was then sintered via SPS method [30] using HP D 5 type equipment (manufactured by FCT Systeme GmBH, Effelder-Rauenstein, Germany). The powders were placed in a graphite die (inner diameter of 20 mm), then uniaxially pressed at 63 MPa, and heated to the sintering temperature at a heating rate of 100 °C/min. A graphite sheet, 0.5 mm in thickness, was inserted between the raw material powder and graphite die to prevent scuffing of the punch and to ease the extraction of the sintered sample from the matrix. The graphite die was also wrapped in carbon blankets to minimise heat loss during sintering. The samples were sintered in the temperature range from 1300 °C to 1600 °C, for 4 min in Ar. The SPS sintered compacts were disk-shaped with dimensions of approx. 20 mm in diameter and 5 mm in height.

### 2.3. Samples Preparation

Regardless of the sintering technique used—either SPS or HPHT—all sintered compacts were ground to remove residual graphite which was necessary for accurate density measurements. This grinding also ensured the parallelism of the frontal surfaces of the samples, a crucial factor for obtaining precise Young’s modulus measurements using the ultrasonic method.

Samples designated for hardness measurements and microstructural studies were additionally subjected to polishing with use of diamond suspensions (with particle sizes of 9, 3 and 1 μm) and colloidal SiO_2_ solutions (with a particle size of 0.4 μm) to achieve a mirror-like finish.

HPHT-sintered samples intended for cutting tests were ground to create ISO cutting inserts with the RNGN 0903 T01220 geometry, which measures 9.53 mm in diameter and 3.18 mm in height. From SPS-ed materials, ISO inserts with the RNGN 1204 T01220 geometry, measuring 12.7 mm in diameter and 4.76 mm in height, were produced through grinding operations.

### 2.4. Testing Methods

The densities of the sintered samples were measured via the Archimedes method while their theoretical densities were calculated by applying the rule of mixtures and assuming densities presented in the Table 1. The Young’s modulus values of the composites were determined via the ultrasonic wave transition method by using an ultrasonic flaw detector (Epoch III, Panametrics, Waltham, MA, USA) to measure the velocities of the ultrasonic sound waves passing through the material. The velocities of the transversal and longitudinal waves were determined as ratios of the sample thickness to the relevant transition time.

The hardness was determined by the Vickers indentation method by applying loads of 9.81 N (1 kg) using a FLC-50VX hardness tester (Future Tech, Kawasaki, Japan). For each sample, five indentations were made.

The microstructure observation was performed on three cBN-TiN-Ti_3_SiC_2_ composite samples sintered by HPHT at temperatures: 1700, 1900 and 2000 °C, using Transmission Electron Microscope Tecnai G2 F20 (FEI Company, Hillsboro, OR, USA) (2000 kV) equipped with HAADF detector intended for scanning-transmission technique (STEM). Each sample was subjected to a preliminary analysis of the microstructure and then the chemical composition of the samples in the micro-areas was analysed and presented in elemental distribution maps.

For AZW sinters, the microstructure analysis was performed using Scanning Electron Microscope JSM-6460LV (JEOL, Tokyo, Japan) equipped with an EDS spectrometer INCA X-act Energy 350 from Oxford Instruments (Abingdon, UK).

Cutting properties of the insert were tested during longitudinal turning. Machining was carried out on an CNC lathe-milling centre type NL2000SY (Mori Seiki, Tokyo, Japan) with 18.5 kW main drive motor power. The workpiece was an Inconel 718 alloy (Bibus Metals, Dabrowa, Poland). The cutting speed was 200 m/min for superhard composites and 90 m/min for CMCs. RNGN 0903 T01220 and RNGN 1204 T01220 geometry were used for superhard and CMCs inserts, respectively. The cutting performance of inserts was assessed basing of tool flank wear measured using an optical microscope (parameter VB_C_ according to the PN-ISO 3685:1996 standard [31]). Tool life was defined as the time to achieve a wear value of VB = VB_C_ = 0.3 mm. Cutting tests were carried out for three cutting blades, from which the average operating time of the cutting insert was calculated. The tool life was compared with commercially available composite tested under the same conditions. The wear of the inserts after the cutting tests was examined using a LEXT OLS 4100 (Olympus, Tokyo, Japan) confocal microscope.

## 3. Results and Discussion

### 3.1. Density and Young’s Modulus

Table 2 presents the results of temperature optimisation for superhard composites sintered via the HPHT method. For each sintered sample, density and Young’s modulus were tested, representing a fundamental quality criterion for the further selection of materials with the most suitable properties. For AZW composites, sintering optimisation is shown in Table 3.

The cBN + TiN + Ti_3_SiC_2_ composites reached full compaction regardless of the sintering temperature. Measurement of Young’s modulus showed slight increase with sintering temperature. Such changes in the value of Young’s modulus, despite constant density, may be due to complex phenomena that involve the decomposition of the MAX phase and the formation of new compounds in the composite.

**Figure 4:**
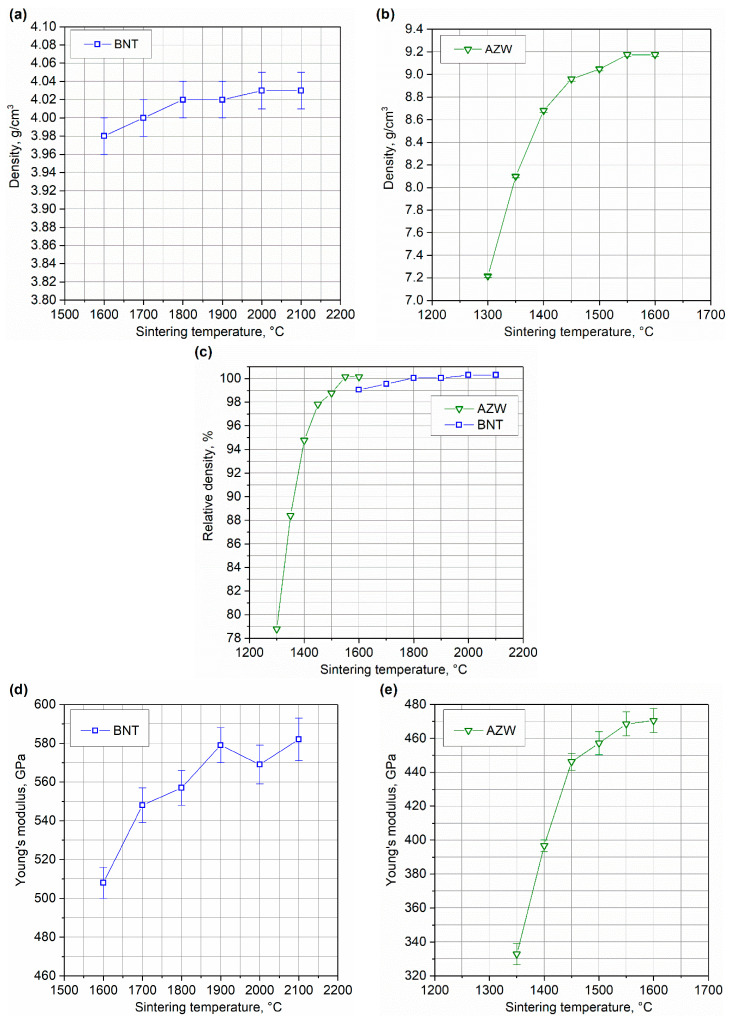
Density (**a**,**b**), relative density (**c**) and Young’s modulus (**d**,**e**) of BNT and AZW composites sintered with HPHT and SPS methods, respectively.

The AZW composite demonstrates completely different behaviour during sintering. This material densifies gradually with increasing temperature, reaching maximum density at a temperature of about 1550 °C (Table 3 and Figure 4b,c). A similar relationship can also be observed for the Young’s modulus of the AZW composite (Figure 4e).

Comparing the densification and changes in Young’s modulus of both composites during sintering (Figure 4), it is evident that at a temperature of approximately 1600 °C, the AZW composite is already fully sintered, while the BNT material has only just begun to sinter. This behaviour clearly results from the phase composition of the two materials; the AZW composite contains alumina, which has a low sintering temperature (~1250 °C under SPS conditions), whereas the BNT composite consists solely of phases that require higher sintering temperatures.

### 3.2. Hardness

**Figure 5:**
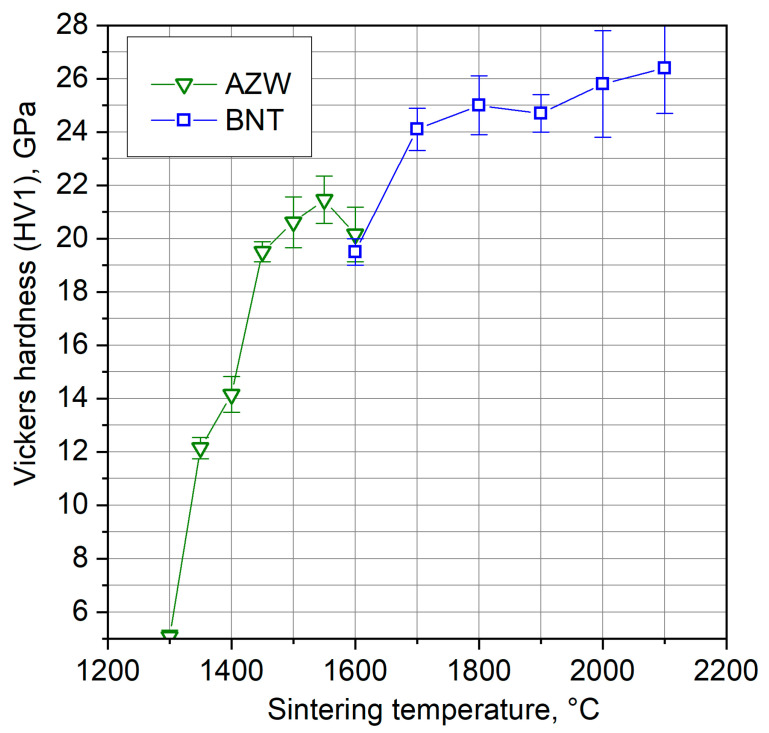
Vickers hardness of BNT and AZW composites sintered with HPHT and SPS methods, respectively.

The Vickers hardness (HV1) results, as shown in Figure 5, indicate that the hardness of both composites changed with sintering temperature.

For the AZW composite, hardness increased significantly with sintering temperatures from 1300 °C to 1450 °C, reaching approximately 20.5 GPa at 1450 °C. Beyond this point, the rate of increase slowed considerably. The maximum hardness for this material, measured at 21.5 GPa, was observed in the sample sintered at 1550 °C. However, increasing the sintering temperature to 1600 °C resulted in a slight decrease in hardness due to grain growth.

In contrast, the BNT composite sintered at 1600 °C exhibited a hardness of about 19.5 GPa, which then jumped to 24 GPa at 1700 °C. At higher temperatures, the hardness of the BNT composite increased more gradually.

Overall, the Vickers hardness of both materials is high. The best sample of the AZW ceramic composite achieves a hardness value greater than 21 GPa, while the BNT composite containing cBN is even harder, reaching more than 26 GPa.

### 3.3. Microstructure and Phase Composition of BNT Superhard Composites

**Figure 6:**
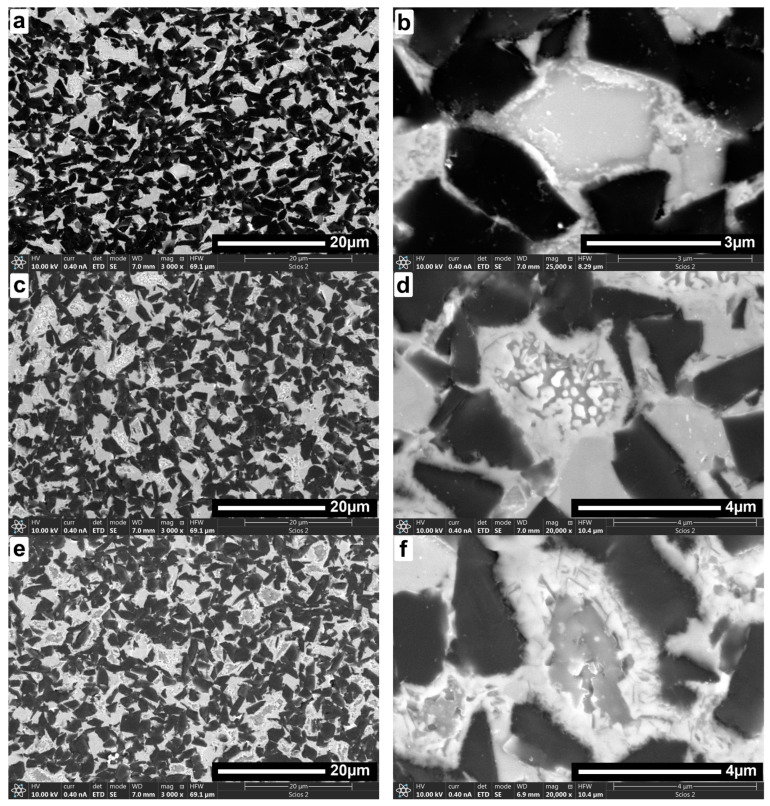
SEM microstructures of BNT superhard composites sintered with HPHT at temperatures: 1700 °C (**a**,**b**); 1900 °C (**c**,**d**); 2100 °C (**e**,**f**).

**Figure 7:**
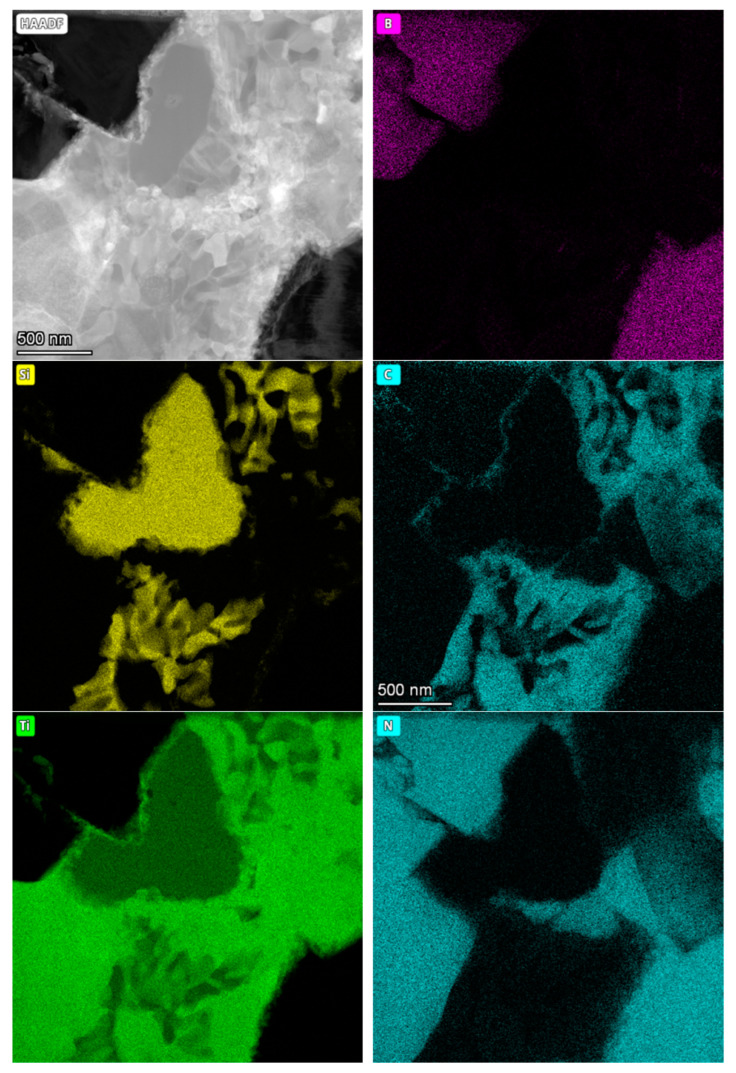
STEM/HAADF image and EDS analysis of BNT composite sintered at 1700 °C.

**Figure 8:**
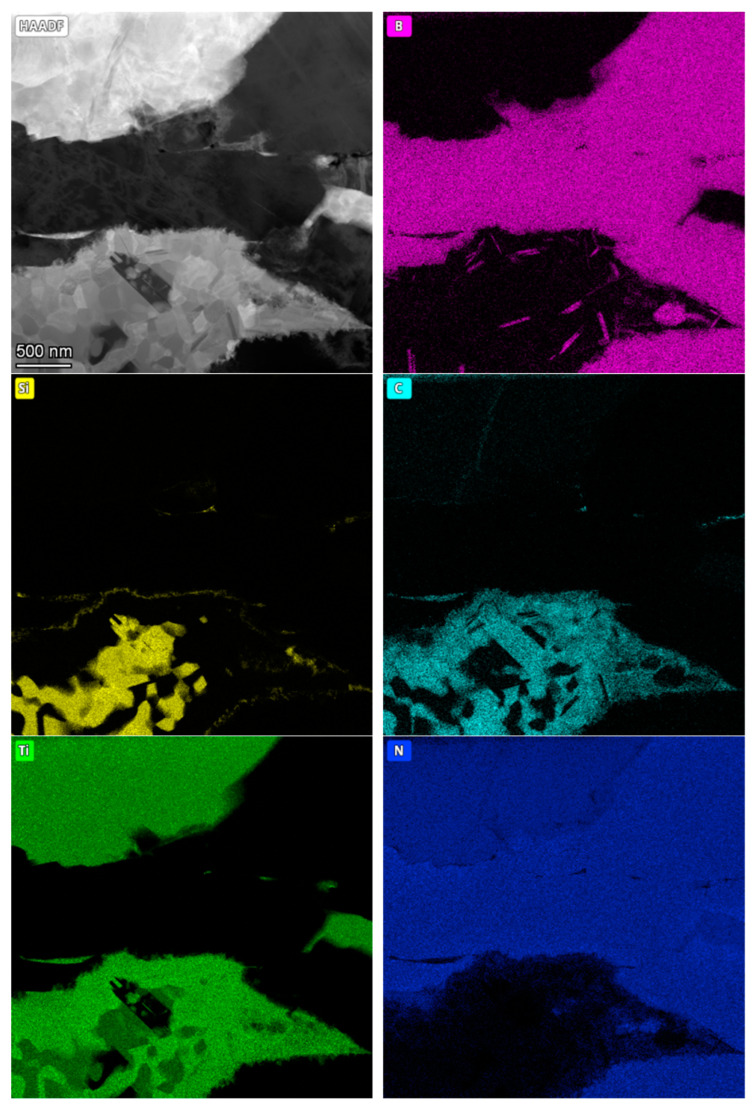
STEM/HAADF image and EDS analysis of BNT composite sintered at 1900 °C.

**Figure 9:**
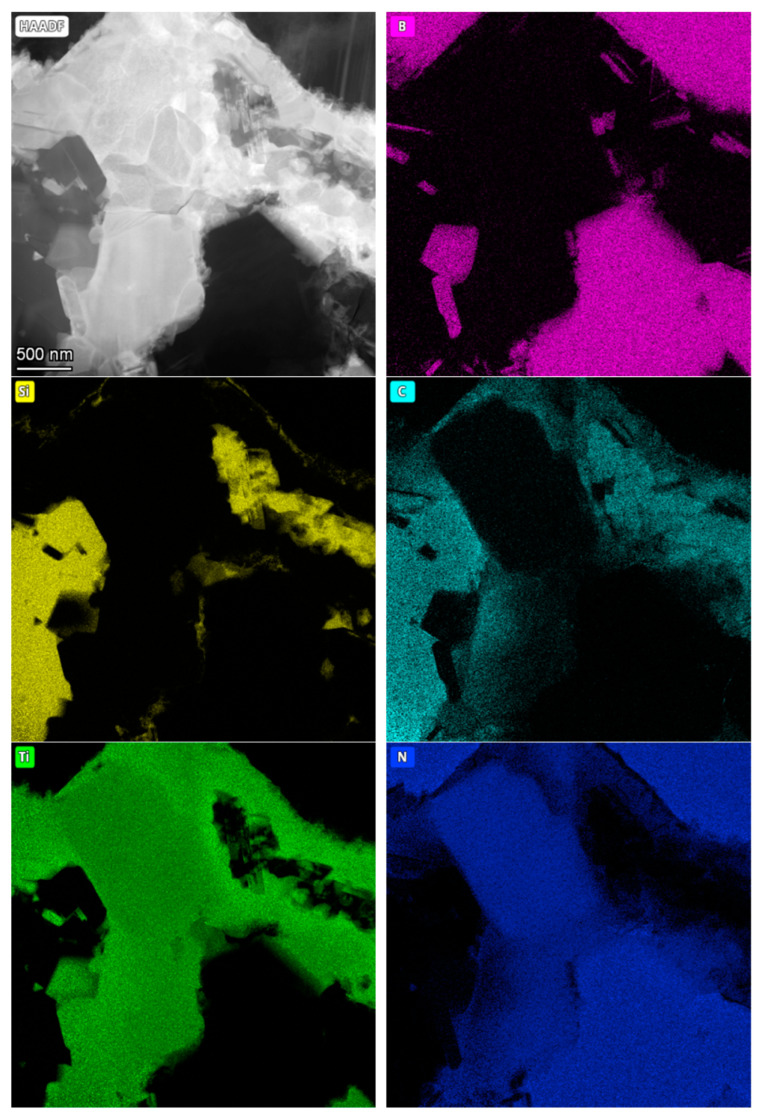
STEM/HAADF image and EDS analysis of BNT composite sintered at 2100 °C.

The following figures show microstructure observations (Figure 6) and EDS maps (Figure 7, Figure 8 and Figure 9) for BNT superhard composites obtained by HPHT sintering. Three representative samples, sintered at 1700, 1900 and 2100 °C, respectively, were selected for the observations in order to present the changes occurring in the microstructure of the composites as the sintering temperature increases.

SEM images (Figure 6) revealed a microstructure consisting of multi-walled cBN grains with sharp edges (dark areas) and a uniformly distributed bonding phase, which can be seen as bright areas. Microstructure observations taken at low magnifications indicate a homogeneous distribution of microstructure components regardless of sintering temperature. Neither the growth nor excessive agglomeration of the individual composite components was observed. The sinters exhibit a bi-modal structure, with large crystallites separated by fine crystalline material. With increasing sintering temperature, diffusion of the components within the bonding phase was observed, resulting in the appearance and growth of a heterogeneous structure initially similar to eutectics, which is particularly evident in higher magnification pictures. As the sintering temperature increased, the diffusion of components was noticeably more intense.

The EDS analysis confirmed the multiphase nature of the sinters. For the composite sintered at 1700 °C, titanium was found throughout the bonding phase, with the intensity decreasing in areas of silicon. Silicon concentrated into characteristic structures, surrounded mainly by carbon- and titanium-containing phases. Nitrogen, in addition to occurring within the BN grains, also appeared in the bonding phase, partially overlapping with the occurrence of titanium which may indicate the presence of a TiN phase.

For the composite sintered at 1900 °C, it was observed that boron, other than appearing within the cBN grains, formed fine needle-like structures within the bonding phase. Carbon, as in the previous sample, was detected around the silicon. Titanium was identified throughout the bonding phase, while nitrogen was not uniformly present in the bonding phase, its intensity dropping almost completely in the areas of silicon and carbon occurrence.

At the highest sintering temperature of 2100 °C, boron was also found to be present within the bonding phase, which may indicate the presence of a TiB_2_ phase. Silicon, which in previous composites had partially overlapped with titanium, diffused intensively to form structures clearly separated from the other components, containing only negligible amounts of carbon. Nitrogen, as in previous composites, was present within the cBN grains and the bonding phase together with titanium (TiN phase).

The intensive diffusion of the composite components, specifically silicon, which occurred as the sintering temperature increased, enables to conclude that under the applied sintering conditions an intensive phase transformation took place within the bonding phase, which initially consisted of TiN and Ti_3_SiC_2_ phases. As is known, Ti_3_SiC_2_ phase is unstable under elevated pressure and temperature, therefore its thermal decomposition was expected under the applied sintering conditions. It can be also concluded that mentioned decomposition initiated in situ the formation and evolution of a multicomponent bonding phase. It is highly likely that SiC, TiSi_2_, TiN, TiC and TiB_2_ phases are present; however, further investigations are necessary to confirm these suppositions. Specifically, identifying the phases in micro-areas using electron diffraction methods would provide more definitive results.

**Figure 10:**
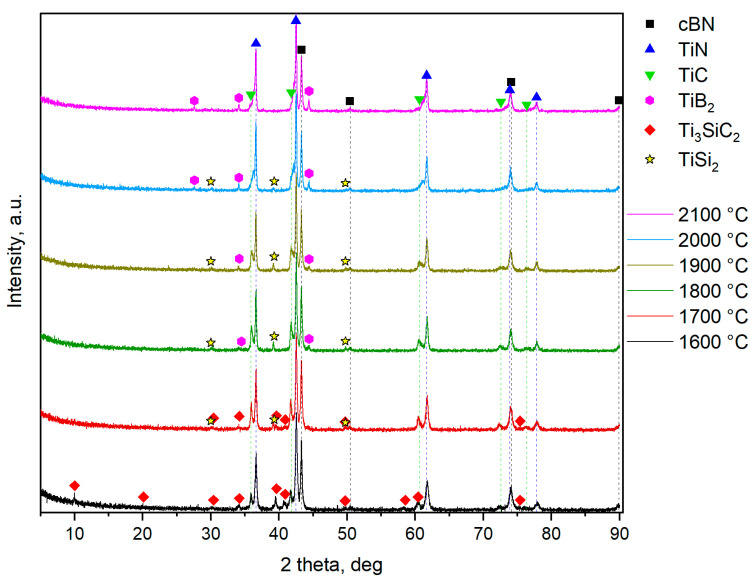
XRD patterns of BNT composite sintered at temperatures from 1600 to 2100 °C.

In the present study, only X-ray diffraction was conducted, which gives averaged information from larger areas (Figure 10).

X-ray diffraction confirmed the evolution of the phase composition of the BNT composite with increasing sintering temperature. At 1600 °C, only the phases present in the initial powder mixture were observed: cBN, TiN, Ti_3_SiC_2_ and TiC. Among these, cBN, TiN and TiC were consistently present in the composite throughout the entire temperature range considered (Figure 10, dashed lines). The proportion of TiN to TiC changed slightly with temperature, which could be attributed to the formation of the Ti(C, N) solid solution. As the temperature raised to 1700 °C, the intensity of the Ti_3_SiC_2_ reflections drastically decreased and peaks characteristic of the intermetallic phase TiSi_2_ appeared. At 1800 °C, Ti_3_SiC_2_ completely disappeared, and a new phase TiB_2_ emerged. The intensity of the TiSi_2_ reflections were highest at 1800 °C but decreased with increasing temperature and ultimately vanished at 2100 °C. In the temperature range of 1800 to 2000 °C, the phases cBN, TiN, TiC, TiSi_2_, and TiB_2_ coexisted in varying proportions. As the temperature increased from 1800 to 2100 °C, the intensity of TiB_2_ peaks increased.

Overall, the conclusions drawn from the XRD data align well with those obtained from the EDS maps regarding the formation of new phases, particularly TiSi_2_ and TiB_2_. However, an unexplained discrepancy arises from the absence of silicon-containing phases identified by XRD in the sample sintered at the highest temperature of 2100 °C, which EDS suggests could be silicon carbide.

The cBN–TiN–Ti_3_SiC_2_ system undergoes a very complex evolution in the considered temperature range, despite a relatively short HPHT process lasting only 60 s. The reactions under consideration are therefore strongly non-equilibrium from a thermodynamic perspective. The application of longer dwell times would likely lead to a different phase composition and microstructure. This opens the door for further detailed studies of this system under HPHT conditions.

Due to the decomposition of the MAX phase, it becomes impossible to directly utilize its benefits, especially the metal-like properties such as high plasticity, to create a synergistic effect when combined with superhard materials. However, conducted research indicates that the decomposition of Ti_3_SiC_2_ results in phases that effectively serve as a binding phase in the BNT composite. A thorough investigation of the influence of the phase composition of the BNT composite depending on the HPHT sintering temperature on the cutting properties of this material is planned for future studies.

### 3.4. Microstructure and Phase Composition of AZW Ceramic Composites

**Figure 11:**
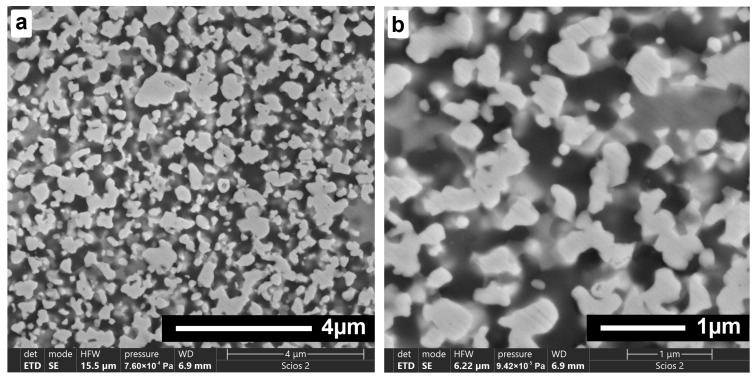
SEM microstructures of AZW composite sintered with SPS at 1550 °C, magnifications: ×20 k (**a**), ×50 k (**b**).

**Figure 12:**
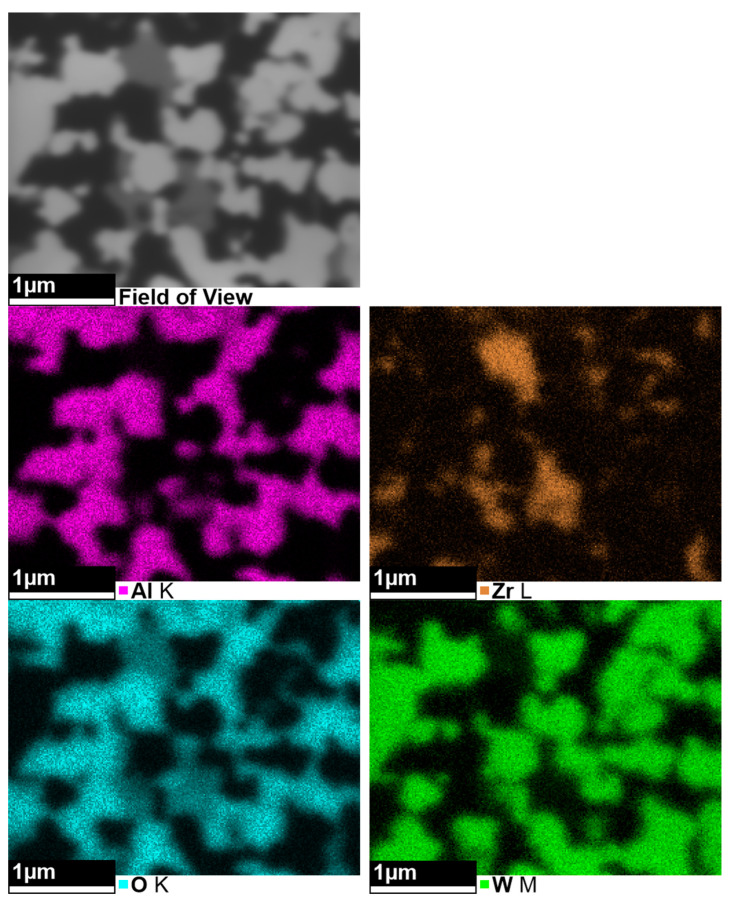
SEM image and accompanying EDS maps of AZW composite sintered at 1550 °C.

The microstructure of the AZW composite obtained via SPS (Figure 11) is highly homogeneous. The EDS analysis (Figure 12) indicates that the white–grey objects visible in the SEM images are WC grains, grey areas are most likely ZrO_2_ and dark grey regions correspond to Al_2_O_3_ phase. The significant phase contrast resulting from the differences in atomic numbers of the elements in the AZW material causes the oxide matrix observed in the scanning electron microscopy images to appear semi-transparent. As a result, the WC particles are also visible beneath the surface of the microsection, which give the impression of three-dimensionality. This pseudo 3D image confirms mutually interpenetrating carbide and oxide phases forming a “duplex microstructure”.

**Figure 13:**
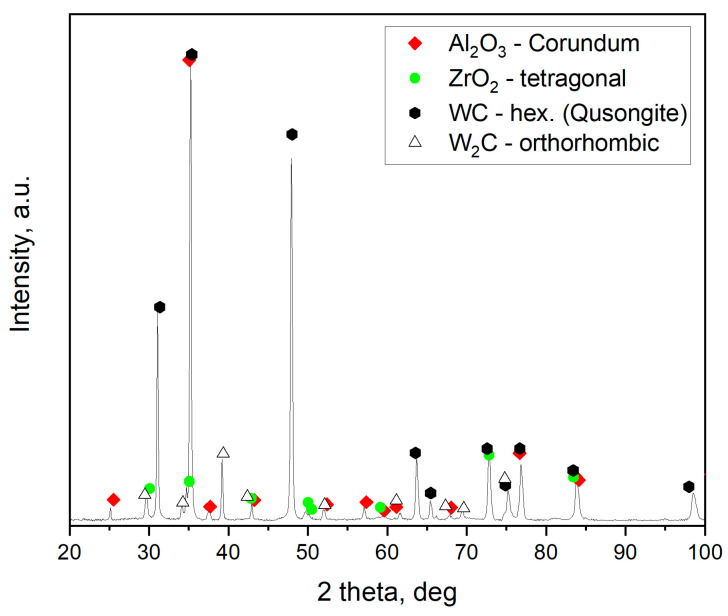
XRD pattern of AZW composite sintered at 1550 °C.

Figure 13 shows an XRD analysis of the AZW composite sintered at 1550 °C. Three initial phases were identified in this sample: Al_2_O_3_, ZrO_2_, and WC. Additionally, tungsten carbide with W_2_C stoichiometry was present in the composite. It should be noted that a small amount of W_2_C was included in the starting powder used to prepare the AZW mixture. A certain amount of W2C could also arise as a result of the reduction of WC during the SPS process. In contrast to the BNT material, the phase composition of the AZW composite does not change significantly within the considered sintering temperature range.

### 3.5. Cutting Tests

**Figure 14:**
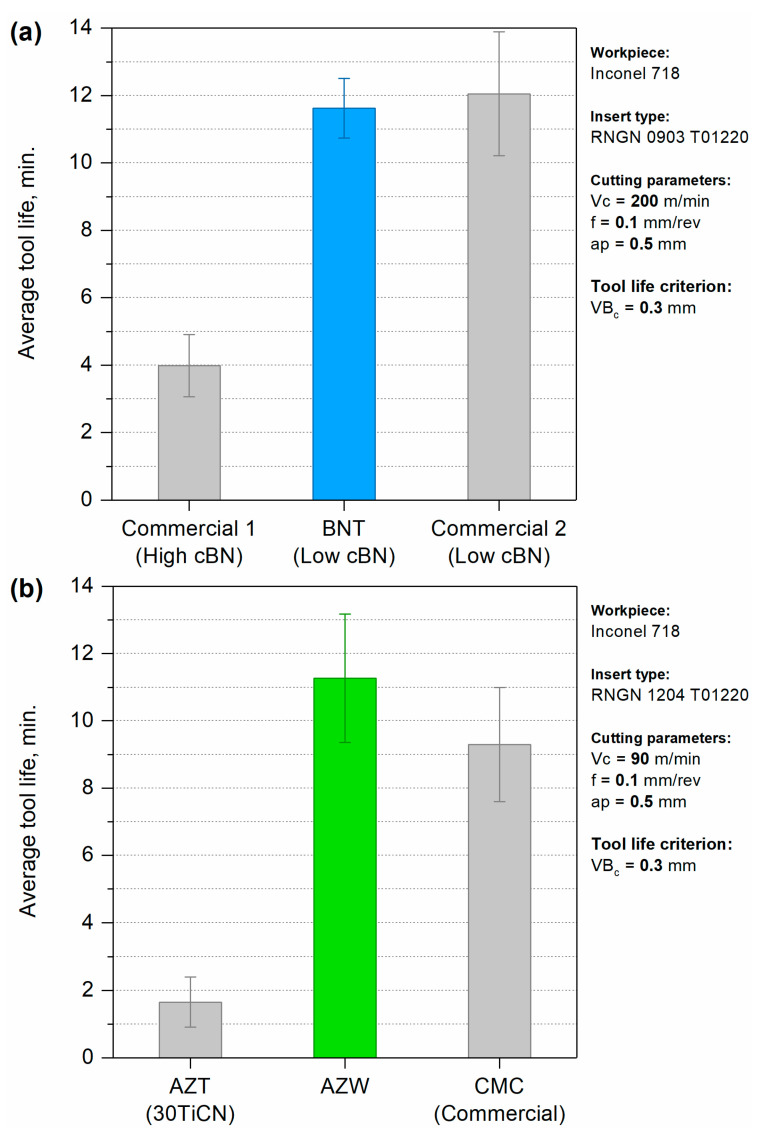
Average tool life of RNGN cutting inserts made from investigated composites when turning Inconel 718 alloy: BNT compared with commercial superhard cBN composites (**a**), AZW compared with other advanced ceramics (**b**).

The results of comparative turning tests of inserts made from BNT (HPHT sintered at 2100 °C) and AZW (SPS-ed at 1550 °C) composites are illustrated in Figure 14. The BNT composite was evaluated against two grades of commercial composites containing cubic boron nitride: a high-cBN type and a low-cBN type. As shown in Figure 14a, the performance of tools cutting Inconel alloy significantly improved with the low-cBN inserts. Both the commercial low-cBN insert and the BNT composite exhibited an average tool life of 11.6 min before reaching critical wear of 0.3 mm at a cutting speed of 200 m/min. In contrast, the high-cBN commercial insert had a much shorter lifespan of only 4 min.

The AZW composite insert was compared with two reference inserts made from advanced ceramics. The first reference, designated as AZT, consisted of oxide-carbide mixed ceramics primarily composed of Al_2_O_3_ and 30 vol% titanium carbonitride Ti(C, N). AZT material was specifically designed for cutting hardened steel and was developed at the Łukasiewicz—Krakow Institute of Technology. The second reference was a commercial advanced ceramic matrix composite (CMC) that contained Al_2_O_3_ reinforced with SiC whiskers.

The turning tests on Inconel 718 demonstrated that the AZW composite achieved an average tool life of 11.3 min before reaching critical wear of 0.3 mm at a cutting speed of 90 m/min. In comparison, the average tool life for the AZT and CMC references was below 2 min and approximately 9 min, respectively. These results indicate that while the AZT reference sample performs good when cutting steel, it does not yield favourable results when machining Inconel. Conversely, the AZW composite investigated in this study shows comparable performance to, or even slightly outperforms, advanced commercial SiC whisker-reinforced ceramics recommended for cutting nickel superalloys.

**Figure 15:**
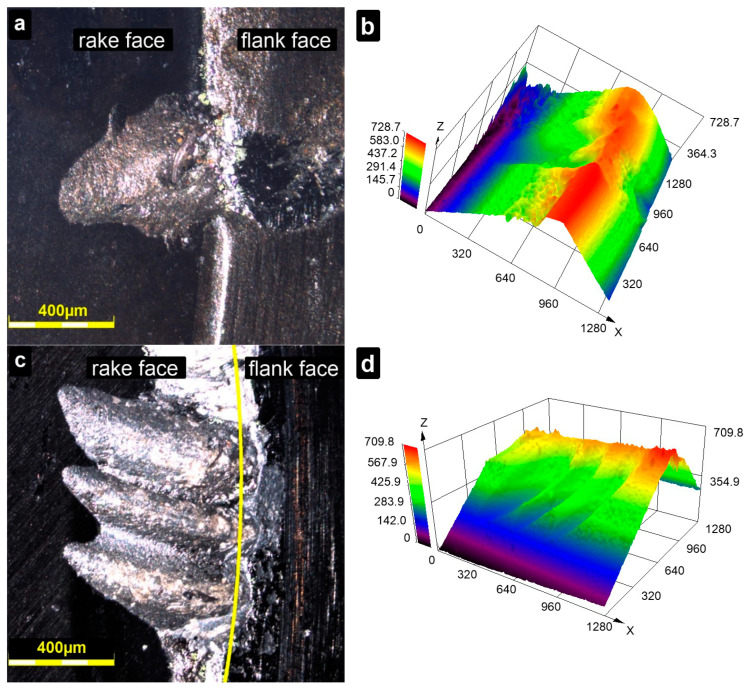
Cutting edges of investigated inserts after turning tests: 2D images (**a**,**c**) and 3D topography maps (**b**,**d**) of BNT insert (**a**,**b**) and AZW insert (**c**,**d**); confocal microscope.

After conducting the cutting tests, the wear on the inserts was analysed using a confocal microscope. This method provided a detailed visualization of the wear characteristics, offering more insights than simply measuring the average flank wear width according to the PN-ISO 3685:1996 standard. As illustrated in Figure 15, both BNT and AZW inserts exhibited wear on both the flank and the rake face, with the wear on the rake face being significantly more pronounced. The observed rake wear is complex in nature, with crater wear being the predominant form. Crater wear develops on the rake face due to high temperatures at the tool-chip interface. Additionally, the chemical affinity between the tool material and the workpiece can exacerbate this wear, resulting in material loss from the insert. Such wear patterns are often encountered on cutting tools after machining of Inconel alloys [1,32].

In the case of the BNT insert, craters are accompanied by some chipping of material. In contrast, the craters occurred in AZW compact exhibit an elongated shape, suggesting that notch wear mechanisms were also at play. This might include abrasion from γ″ (Ni_3_Nb) and γ′ (Ni_3_(Al,Ti)) precipitates present in the Inconel structure, as well as thermal effects [2].

The BNT material containing superhard cBN phase and the AZW ceramic composite were compared in presented work individually to the respective reference materials. Also, different cutting speeds of 200 and 90 m/min were used for the superhard and the ceramic material, respectively (the feed rate of 0.1 mm/rev and depth of cut of 0.5 mm were the same for both insets). For such test parameters, the average lifetimes of inserts made from both materials were very similar: 11.6 and 11.3 min for BNT and AZW, respectively. To eliminate cutting speed as a variable and to compare the cutting efficiency and economy of BNT and AZW materials with each other, the Material Removal Rate (MRR) index was used (Equation (1)):

MRR = ap∙f∙Vc(1)

**Figure 16:**
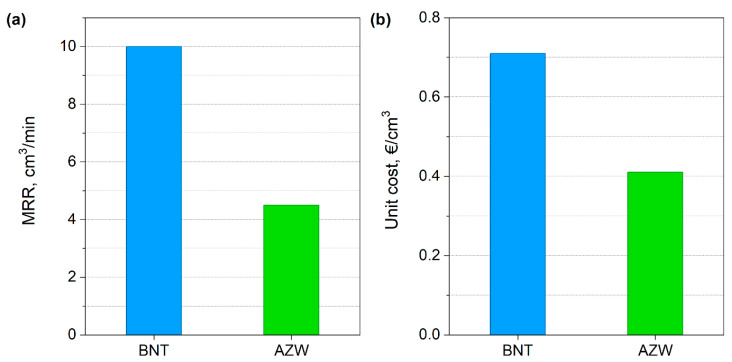
The comparison of MMR index (**a**) and unit cost of material removing (**b**) for BNT and AZW inserts.

This index allows to calculate the volume of material removed per time unit during machining operations such as milling, turning, drilling, and grooving. At the given test parameters, the MRR for index for BNT and AZW inserts was 10 and 4.5 cm^3^/min, respectively (Figure 16a). Assuming that the unit cost of a tool made from BNT material is EUR 82 and for AZW material is EUR 24 (assumption based on the prices of selected commercial grades, as the actual manufacturing costs of single samples obtained at a research unit are difficult to estimate and are certainly much higher than the costs in optimized serial production) and knowing the average lifespan of both inserts, the calculated cost of removing of 1 cubic centimetre of Inconel 718 with BNT is ~EUR 0.71 while using AZW is ~EUR 0.47 (Figure 16b).

The comparative tests conducted lead us to conclude that the BNT superhard composite can be classified as “premium”, because its use is associated with higher costs but also gives significantly greater machining efficiency. In contrast, while AZW composite ranks at the top of its class among advanced ceramic materials, it offers lower efficiency compared to the superhard material. However, using inserts made from AZW results in a significantly lower unit cost for removing a unit volume of the workpiece. These factors should be considered when designing tool materials.

## 4. Conclusions

Two types of cutting tool materials intended for machining Inconel 718 superalloy were prepared and investigated. The first material was a superhard composite sintered from the cBN-TiN-Ti_3_SiC_2_ system using the HPHT method, while the second was a ceramic composite sintered from the Al_2_O_3_–ZrO_2_–WC system using SPS.

For the cBN-TiN-Ti_3_SiC_2_ system, many reactions occurred during sintering due to the highly reactive Ti compounds forming the bonding phase, resulting in a final phase composition of the BNT superhard composite that differed significantly from the initial composition. In contrast, the phase composition of the AZW ceramic composite remained consistent before and after sintering.

The relative density of the samples sintered at optimized temperatures in both cases was nearly 100%. However, it should be noted that for the BNT composite, the reference values are based on the initial composition, so the relative density of this material should be considered indicative only. The Young’s modulus values of 580 GPa for BNT and 470 GPa for AZW, along with hardness values of 26 GPa for BNT and 21 GPa for AZW, indicate that both materials perform well within their respective classes.

Inserts made from both materials demonstrated excellent cutting properties, with average tool lifetimes comparable to top commercial products in their classes. Comparing the inserts with each other, it can be seen that the insert made from BNT superhard composite exhibited better performance, showing a material removal rate of 10 cm^3^/min, while the insert made from AZW ceramic composite achieved an MRR of 4.5 cm^3^/min. The unit cost of removing workpiece material with the BNT insert was higher (~EUR 0.71/cm^3^) than that when using the AZW insert (~EUR 0.47/cm^3^).

The cutting inserts developed in this work can effectively meet market demands, whether the focus is on achieving the highest machining efficiency or on cost considerations. However, to fully harness the potential of these materials, systematic research is required. This includes further optimization of the HPHT sintering parameters for the BNT composite, as well as conducting cutting tests on both BNT and AZW composite inserts during the machining of various hard-to-cut alloys using different combinations of cutting parameters.

## Figures and Tables

**Table 1:** Densities and Young’s moduli of the starting materials.

	cBN	TiN	Ti_3_SiC_2_	Al_2_O_3_	ZrO_2_	WC
Apparent density, g/cm^3^	3.48	5.39	4.52	3.98	6.05	15.7
Young Modulus, GPa	909	465	325	400	219	705

**Table 2:** Density and Young’s modulus for BNT composites after HPHT sintering.

Sintering Temperature, °C	Density, g/cm^3^	Relative Density, %	Young’s Modulus, GPa
1600	3.98 ± 0.02	99.1	508 ± 8
1700	4.00 ± 0.02	99.5	548 ± 9
1800	4.02 ± 0.02	100	557 ± 9
1900	4.02 ± 0.02	100	579 ± 9
2000	4.03 ± 0.02	99.8	569 ± 10
2100	4.03 ± 0.02	100	582 ± 11

**Table 3:** Density and Young’s modulus for AZW composites after SPS sintering.

Sintering Temperature, °C	Density, g/cm^3^	Relative Density, %	Young’s Modulus, GPa
1300	7.22 ± 0.02	78.8	– *
1350	8.10 ± 0.02	88.4	333 ± 6
1400	8.68 ± 0.02	94.8	397 ± 3
1450	8.96 ± 0.02	97.8	446 ± 5
1500	9.05 ± 0.02	98.8	457 ± 7
1550	9.17 ± 0.02	100	469 ± 7
1600	9.17 ± 0.02	100	471 ± 7

* unmeasurable with ultrasonic method.

## Data Availability

The original contributions presented in this study are included in the article. Further inquiries can be directed to the corresponding author.
